# Elucidating the effect of tumor and background region-of-interest selection on the performance metrics used to assess fluorescence imaging

**DOI:** 10.1117/1.JBO.30.4.046004

**Published:** 2025-04-02

**Authors:** Augustino V. Scorzo, Caleb Y. Kwon, Rendall R. Strawbridge, P. Jack Hoopes, David W. Roberts, Scott C. Davis

**Affiliations:** a Dartmouth College, Thayer School of Engineering, Hanover, New Hampshire, United States; b Dartmouth College, Geisel School of Medicine, Hanover, New Hampshire, United States; c Norris Cotton Cancer Center, Dartmouth-Hitchcock Medical Center, Lebanon, New Hampshire, United States

**Keywords:** contrast agents, performance metrics, fluorescence cryotomography, fluorescence-guided surgery, glioma

## Abstract

**Significance:**

The development of fluorescent contrast agents for fluorescence-guided surgery is rapidly growing with many agents being designed for tumor visualization. Although efforts have been made to standardize the sensitivity of imaging system detection methods for these contrast agents, guidelines to evaluate tumor contrast agent performance, especially the selection of tumor and background regions of interest (ROIs), differ widely across studies. We examine how systematically changing tumor and background ROIs affects common metrics of contrast agent performance.

**Aim:**

We aim to elucidate the influence of changing tumor and background brain regions of interest on fluorescent contrast agent performance.

**Approach:**

Mice with orthotopic brain tumors were administered a non-targeted fluorescent contrast agent 40 min prior to sacrifice and then imaging of the specimen using whole-body fluorescence cryotomography. The reconstructed 3D fluorescence volumes were then used to compute contrast and diagnostic performance metrics [tumor-to-background brain ratio (TBR), contrast-to-noise (CNR), and area under the receiver operating characteristic curve (AUC)] while systematically varying tumor and normal brain ROIs.

**Results:**

ROI selection had a significant impact on the reported values of metrics used to evaluate fluorescence imaging strategies. The use of contralateral background ROIs, commonly used in the field, produced elevated and favorable performance metric values. These metrics decreased as background ROIs approached regions adjacent to the tumor boundary. TBR changed by a factor of 5, CNR by a factor of 7, and AUC by over 10%, largely depending on the proximity of the background region to the tumor.

**Conclusions:**

Background ROI selection has a significant impact on the performance metrics commonly used in the field. Future studies should carefully select ROIs relevant to the application and include clear descriptions of these regions.

## 1 Introduction

Fluorescence imaging is a widely used technique that can provide sensitive and real-time visualization of diverse biological processes with high spatial resolution. Over the past several decades, numerous fluorescence imaging techniques have been successfully integrated within the clinical setting to assist in evaluating the structure and function of tissues of interest.^1^^–^^3^ One of the most successful and rapidly growing clinical examples is fluorescence-guided surgery (FGS) which is now a widely adopted surgical method. In this approach, fluorescent contrast agents are administered to a patient to provide real-time imaging of tissue structures within the surgical field, including tumors and blood vessels to guide surgical decision-making, and FGS is also being investigated for additional anatomical structures.^2^^–^^4^ The majority of fluorescent contrast agents, whether currently under development or already approved by the Food and Drug Administration, are designated for tumor visualization and either readily accumulate within tumors or otherwise target specific features overexpressed in or on tumor cells.^5^^–^^18^ The goal of these fluorescent agents is to provide tumor-specific contrast against background or normal surrounding tissues during the surgical procedure to assist in resection.

The visualization of these fluorescent contrast agent distributions during surgical procedures is enabled by fluorescent imaging devices and many efforts to standardize sensitivity assessment for these systems, especially for multi-institutional clinical trials, are currently ongoing.^1^^,^^19^^–^^24^ Although strides have been made to standardize imaging system sensitivity, the standardization of fluorescent contrast agent performance reporting is lacking. Although metrics including tumor-to-background ratio, contrast-to-noise ratio, and diagnostic accuracy are common metrics used, the definitions of tumor and background tissue regions-of-interest (ROIs) underlying these metrics differ widely across studies. Standard guidelines to select ROIs for fluorescent contrast agent performance metric evaluation are not readily available and current procedures can therefore introduce user selection bias.^25^

Most current approaches assess bulk tumor contrast against normal tissue but do not typically evaluate contrast agent performance at, or near, tumor margins. The performance of these contrast agents at the tumor margins will play a critical role in FGS as margin status is a significant prognostic factor for disease recurrence.^26^^–^^30^ Therefore, the investigation of near-tumor margin results in performance metric reporting will provide an important consideration when assessing contrast agent distributions and can enhance existing techniques. Previous studies in phantoms have helped elucidate the impact ROI selection can have on evaluating contrast agent performance^31^^,^^32^ and Dijkhuis et al. recently reported on a semi-automated approach toward standardizing ROI selection on clinical specimens.^33^ Yet, to date, a systematic study that examines the relationship between common performance metrics and a wide range of ROI selection strategies in animal models has not been reported.

In this study, we conducted a systematic evaluation of the effect of ROI selection on fluorescent contrast agent performance metrics. Specifically, we used high-resolution 3D fluorescence cryotomography image volumes of a non-targeted contrast agent administered to mice with orthotopic brain tumors to evaluate commonly used performance metrics while varying tumor and background ROIs over a wide range of configurations. The contrast agent administered in this study was tetramethylrhodamine conjugated to a 1-kDa mPEG chain (TMR-PEG1k) which has recently shown promise in animal models as a fast-acting and persistent agent for fluorescence-guided surgery.^16^ The cryotomography imaging strategy used herein enables the analysis to consider the true fluorescence distribution throughout the tissue, without the influence of confounding factors such as whole blood pooling on the surface, sub-surface fluorescence, and factors specific to individual surgical imaging configurations. Tumor-to-background ratio (TBR), contrast-to-noise ratio (CNR), and the area under the receiver operating characteristic curve (AUC) were computed and compared for each ROI configuration to help establish guidelines for standardization.

## 2 Materials and Methods

### 2.1 Experimental Design

This study was designed to evaluate how changes in tumor and background ROIs affect contrast and diagnostic performance metrics in fluorescence imaging. Mice (N=5) with GFP-labeled orthotopic brain tumors were administered a promising new non-targeted contrast agent (TMR-PEG1k)^16^ and then euthanized at a predetermined time point. The whole specimens were then imaged using 3D fluorescence cryotomography to produce high-resolution volumes of the distribution of GFP (for ground truth) and TMR-PEG1k fluorescence. A variety of tumor and background ROI pairs were defined, and for each pair, TBR, CNR, and AUC were computed and compared.

### 2.2 Animal Models

All studies were performed in accordance with protocols approved by the Institutional Animal Care and Use Committee at Dartmouth College. In this study, 7 to 10-week-old female nude mice (Charles River Laboratory, Wilmington, Massachusetts, United States) underwent intracranial inoculation with $10^{6}$ U87 GFP-expressing glioma cells (Neuromics, Minneapolis, Minnesota, United States) using a previously described surgical procedure.^34^ Following surgery, animals were placed on a chlorophyll-free diet (MP Biomedicals, Irvine, California, United States) to limit tissue autofluorescence for imaging. Contrast-enhanced MRI was used to monitor tumor growth until a tumor size of ∼2  mm was reached, and the animal was put on study.

### 2.3 Imaging Agent and Animal Preparation

The non-targeted agent TMR-PEG1k (CreativePEGWorks, Durham, North Carolina, United States), a tetramethylrhodamine conjugated to a 1-kDa methoxy polyethylene glycol (mPEG) chain was prepared from aliquots reconstituted in phosphate buffered saline and kept at −20°C. Before use, TMR-PEG1k concentration was confirmed with absorption spectrometry. After tumor presence was confirmed using CE-MRI, mice were administered 42.1 nmol of the agent in the tail vein 40 min before euthanasia. At the designated time point, animals were euthanized and prepared for whole-body fluorescence cryotomography.

### 2.4 Whole-body 3D Fluorescence Cryotomography and Histopathology

**Fig. 1:**
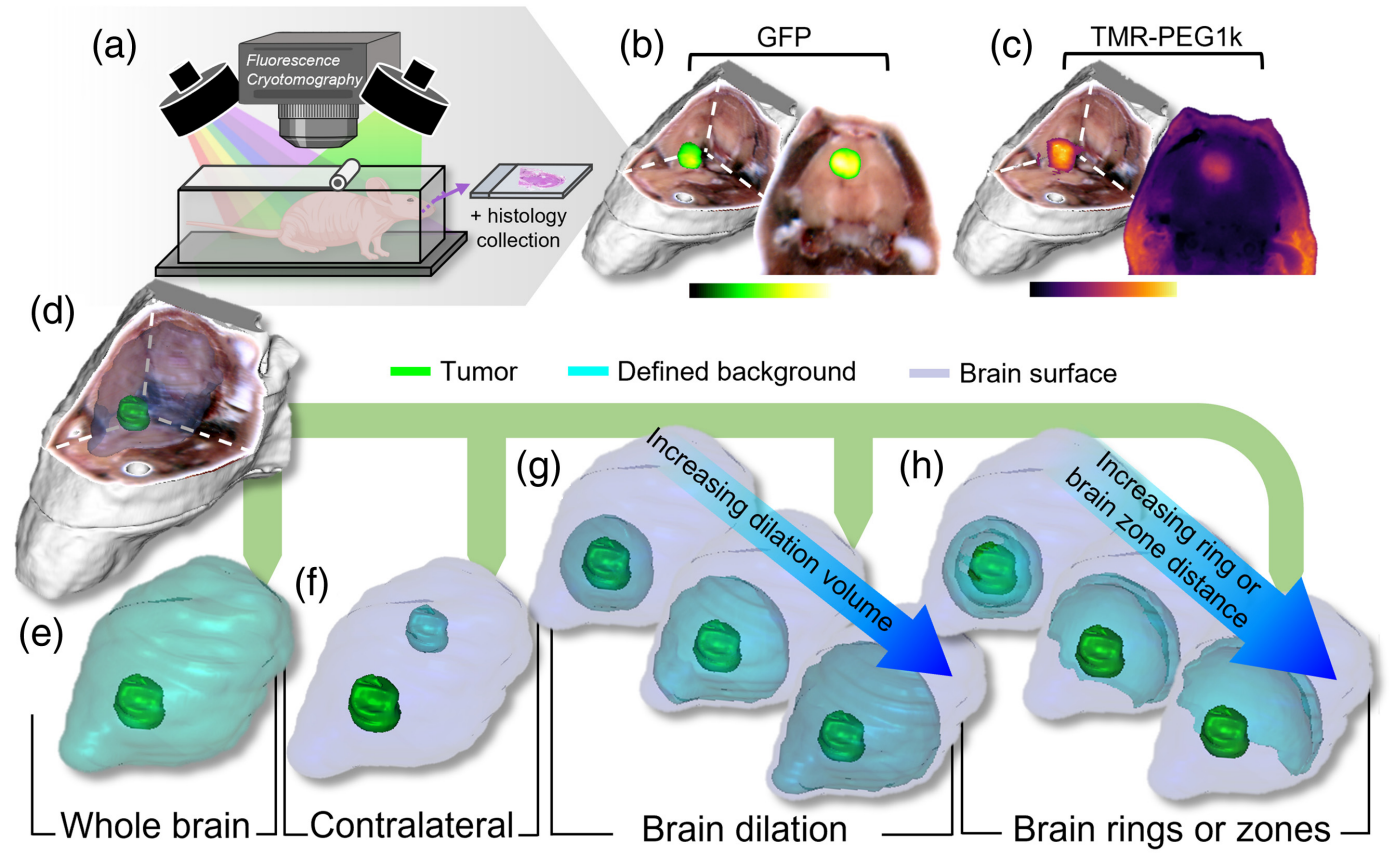
(a) Illustration of the fluorescence cryotomography system which automatically sections and images a specimen block. (b) Representative GFP and (c) TMR-PEG1k fluorescence cryotomography volumes. (d) RGB 3D rendering of a mouse head with tumor and normal brain surfaces. The background brain used for assessing contrast metrics was defined using one of five methods: (e) whole brain, (f) contralateral brain, (g) brain dilation method, and (h) brain ring and brain zone methods.

The large-specimen 3D fluorescence cryotomography system has been described in detail previously^16^^,^^35^ and illustrated in Figs. 1(a)–1(c). This imaging technique applies an automated section-and-image approach to large frozen specimens to produce image stacks through the entire specimen volume. These image stacks can then be visualized and analyzed as high-resolution 3D volumes.^16^^,^^36^^–^^42^ In this study, three LED sources (Mightex, Toronto, Ontario, Canada) were used: (1) a white light RGB imaging, (2) a 530-nm LED with a 550-nm short pass filter for TMR-PEG1k fluorescence imaging, and (3) a 470-nm LED with a 475-nm short pass filter for GFP fluorescence imaging. Specimens were sectioned at 100  μm. The image processing routine is described elsewhere and involves next-image correction to correct for subsurface fluorescence from the underlying specimen block for each image, facilitating analysis of the true fluorescence distribution.^43^^,^^44^ To limit spectral overlap between TMR-PEG1k and GFP, emission from the 530-nm LED channel was analyzed between 620 and 650 nm, whereas the 470-nm LED channel was analyzed between 510 and 530 nm. The final co-registered image volumes were visualized using 3D Slicer.^45^

Once per specimen, the imaging process was paused to collect sections for H&E staining to further establish ground truth, a process which was described previously.^16^ Prepared slides were scanned on an Odyssey M (LI-COR Biosciences, Lincoln, Nebraska, United States) at 10  μm resolution and read by a pathologist to delineate the tumor border.^46^ These sections were also co-registered to the corresponding image slice from the cryotomography system using the point based rigid registration tool in 3D Slicer.^47^

### 2.5 Determination of the True Tumor Boundary

The ground truth for the tumor was determined using the GFP volumes and corresponding co-registered H&E sections, as shown in Fig. S1 in the Supplementary Material. First, tumor boundaries were marked manually on the H&E sections. Next, using the corresponding GFP image slice, we scanned GFP fluorescence intensity threshold values and for each value computed the Dice score between the H&E and GFP images. We then selected the threshold value that provided the highest Dice score and applied that threshold to the entire GFP volume to determine the volumetric tumor boundary.

### 2.6 Region of Interest Selection and Analysis

Once the true tumor region was determined using the GFP fluorescence per animal as outlined in Sec. 2.5, a variety of volumetric tumor and background ROIs were defined and used to calculate TBR, CNR, and AUC for analysis. Figure 1 helps illustrate the different ROI configurations examined. Figure 1(d) shows a representative 3D rendering of the RGB volume with the tumor and whole brain surfaces overlayed in green and blue, respectively, for a single animal. The background brain region was defined using one of the following five methods, also depicted in Figs. 1(e)–1(h):

1. Whole brain [Fig. 1(e)]:
  a. Background: entire normal brain volume segmented from the RGB volume with the area of the tumor subtracted.
  b. Tumor: entire true tumor volume (determined using the approach described in Sec. 2.5).
2. Contralateral brain [Fig. 1(f)]:
  c. Background: region within the brain segmented from the RGB volume, approximately equal in volume to the tumor, that is on the opposite hemisphere of the tumor. This region was positioned 1 to 2 mm from the brain surface and avoided the ventricles.
  d. Tumor: entire true tumor volume (determined using the approach described in Sec. 2.5).
3. Brain dilation [Fig. 1(g)]:
  e. Background: iteratively change the defined background brain region by volumetric image dilation in 3D, where the initial background brain region starts as the first 80  μm dilation outward from the tumor boundary into the normal brain (with the tumor region subtracted). The volume of the defined background brain volume then grows with each 80  μm dilation until reaching 4 mm past the tumor boundary into the normal brain. Each brain dilation is constrained within the boundary of the whole brain.
  f. Tumor: the defined tumor volume started at the tumor and normal brain border as a 40-μm-thick shell into the tumor space. The tumor volume shell was then grown toward the tumor center with each 40-μm dilation iteration until the size of the whole tumor volume was reached.
4. Brain rings [Fig. 1(h)]:
  g. Background: similar to the brain dilation technique, but here, volumetric regions are not cumulative. The first brain ring iteration was an 80-μm dilation outward into the normal brain from the tumor mask with the tumor subtracted, creating an 80-μm-thick ring. Instead of growing this ring into a larger volume, additional concentric rings are generated by moving 80  μm farther into the normal brain from the previous ring. In other words, the outer edge of each new ring is 80  μm farther into the brain than the previous ring and the inner edge of the new ring is the previous ring’s outer edge.
  h. Tumor: same as in method 3 above.
5. Brain zones: A previously reported method^48^ included here for comparison. Similar to the brain ring technique but using 400-μm-thick rings representing cumulative zones within the normal brain region. The first zone is the peri-tumoral zone (0 to 400  μm), followed by the near (400 to 800  μm) and far zones (800 to 1200  μm). Clinically, these zones represent regions of diffuse glioblastoma infiltration into the normal brain and can be critical to delineate and evaluate in an effort to achieve maximal safe resection. Here, these brain zones were applied as background ROIs for evaluating the performance metrics of a fluorescent contrast agent for FGS.

These defined tumor and background brain ROIs were then applied to the TMR-PEG1k fluorescence volume for contrast metric assessment. Performance metrics were computed at each iteration of the tumor and background brain for all animals. Boxplots were plotted to show the median with interquartile range and singular points for each animal, and the 95% confidence interval was calculated across a pooled mean where all animals in the group had data points.

## 3 Results

**Fig. 2:**
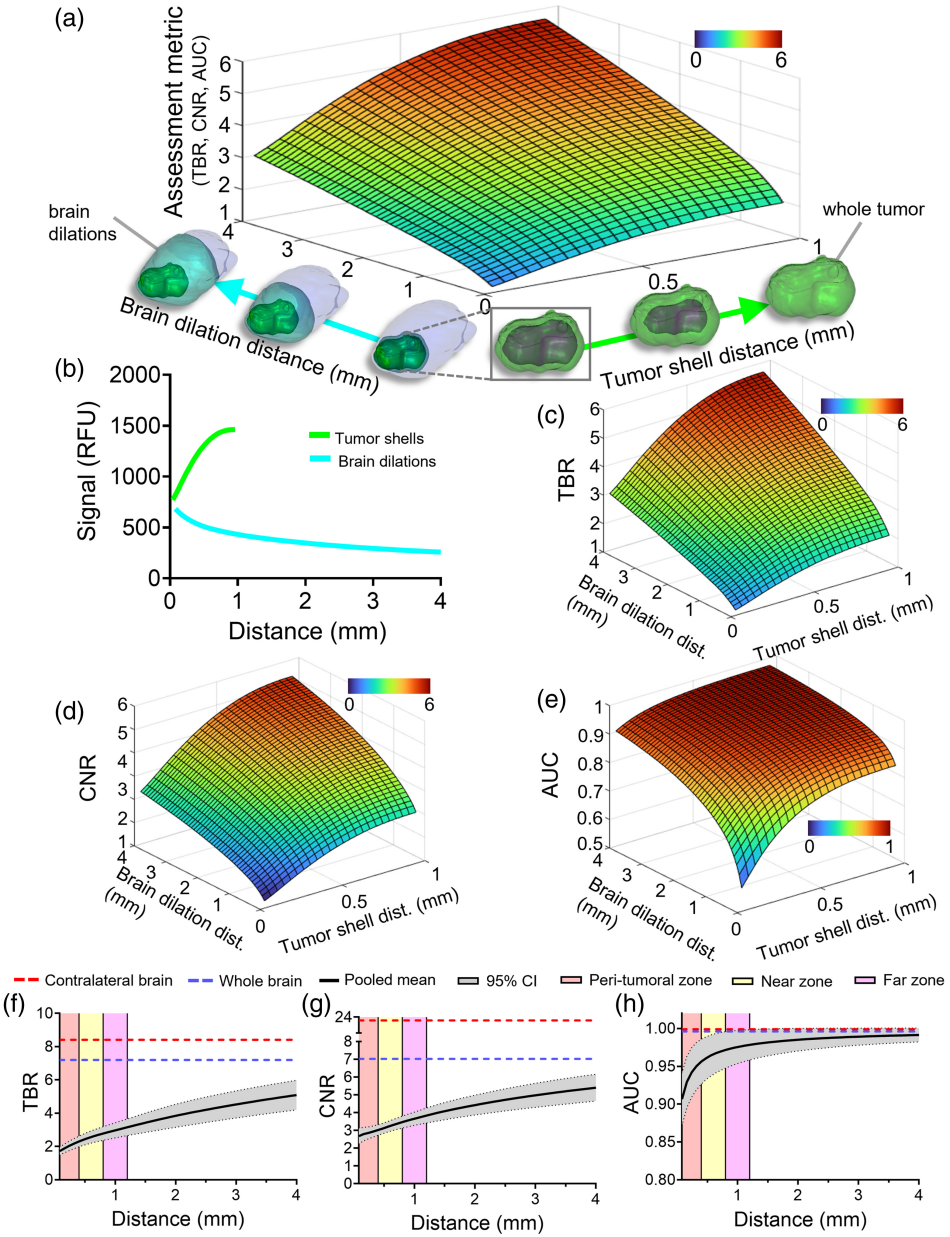
Results for the brain dilation method. (a) Illustrative surface plot of a generic metric as the tumor and background regions are varied for a single animal. (b) Mean fluorescence signal intensity plotted as a function of distance for the tumor shells and brain dilations. (c) Tumor-to-background (TBR) contrast ratio. (d) Contrast-to-noise (CNR) ratio. (e) Area under the receiver operating characteristic curve (AUC). (f)–(h) Contrast metric results for all animals using the whole tumor but increasing the brain dilation distance: (f) TBR, (g) CNR, and (h) AUC. Data for panels (f)–(h) are represented as a pooled mean with a 95% confidence interval. The mean for each contrast metric using the whole brain and contralateral brain as the defined background are represented as dotted lines on the plot and the peri-tumoral, near, and far zones are represented as colored zones.

Figure 2 presents results for the dilation method and compares them with the whole brain and contralateral brain results. Figure 2(a) helps visualize the type of data produced during the analysis for one animal. Specifically, it shows a surface plot of a generic assessment metric (e.g., TBR) as the tumor and background ROIs are varied. The images under the x- and y-axes illustrate how the tumor and background ROIs change, respectively. Specifically, along the x-axis [labeled “Tumor shell distance (mm)”], the defined tumor region is green, and along the y-axis, the defined background brain region is teal. Figure 2(b) presents a plot of the mean TMR-PEG1k fluorescence intensity measured in relative fluorescence units (RFU) of the tumor shells and brain dilation regions for each distance iteration of this representative animal. Inspection of this graph indicates that as more tumor volume is included within the tumor shell (starting from the tumor border and moving toward the tumor core), the mean intensity increases. In addition, as more of the background brain volume is included in the brain dilation, the mean intensity decreases. Specifically, the initially defined tumor shell and brain dilation regions represent only a 1.1× difference in mean intensities, whereas the final iterations display a 5.7× differential.

We then examined how the changing ROIs affect TBR, CNR, and AUC for this animal. Figure 2(c) shows the 3D plot for TBR with the brain and tumor regions changing as outlined in Fig. 2(a). For the brain dilation method, TBR values increased as the volume of the tumor evaluated included more of the tumor core. This result was seen regardless of the defined background brain region. In addition, as the volume of the brain region is increased to include more of the whole normal brain the TBR value also increased irrespective of the amount of whole tumor that was evaluated. Generally, the highest values are seen when the whole tumor and final brain dilation are used; represented by the top red-colored region of the graph (TBR=5.7). The CNR plot displays similar results to the TBR graph where CNR values increase when the defined tumor and brain region volumes are increased [Fig. 2(d)]. Figure 2(e) displays the AUC results for this animal. As compared with the TBR and CR results, AUC values increase rapidly to values near 1 as the respective brain dilation or tumor region volumes increase [Figs. 2(d) and 2(e)].

The results for all animals using the brain dilation method are provided in Figs. 2(f)–2(i). To simplify the plots, the tumor ROI was held constant (whole tumor), whereas the background brain ROI changed. Each panel presents the grouped mean of all animals, herein termed “pooled mean,” with a 95% confidence interval shaded in grey (plots for each animal are provided in Fig. S2 in the Supplementary Material). The pooled mean is displayed up to a dilation distance of 4 mm. The blue- and red-dotted lines represent the pooled mean values using the whole and contralateral brain methods, respectively. In addition, three colored regions appear on the plots which represent the “Brain zones.” The red shaded region is the peri-tumoral zone (0 to 400  μm), the yellow shaded region is the near zone (400 to 800  μm), and the pink-shaded zone identifies the far zone (800 to 1200  μm).

Inspection of Fig. 2(f) indicates that the highest TBR was achieved when the contralateral brain was used as the background, with a mean value of 8.4, followed by the whole brain method (TBR=7.2). Each brain dilation iteration increased the mean TBR values starting from 1.7 at the first 80  μm into the normal brain and reaching a peak mean of 5.1 at 4 mm. Combined CNR results are displayed in Fig. 2(g) and show similar trends to TBR. Specifically, the CNR was the highest when using the contralateral brain with a mean of 20.0 and the whole brain followed with a mean CNR of 7.0. The lowest mean CNR value using the brain dilation method was the first 80  μm at 2.7, whereas the final dilation provided a CNR of 5.4. Figure 2(h) shows the pooled AUC results for the brain dilation method. Using the contralateral brain provided a mean AUC of 0.999, whereas the whole brain provided a mean value of 0.996. Here, the lowest value was again seen when using the first 80-μm dilation (AUC=0.907), and as more of the normal brain volume was included with each iteration, the AUC increased to 0.991 at 4 mm.

**Fig. 3:**
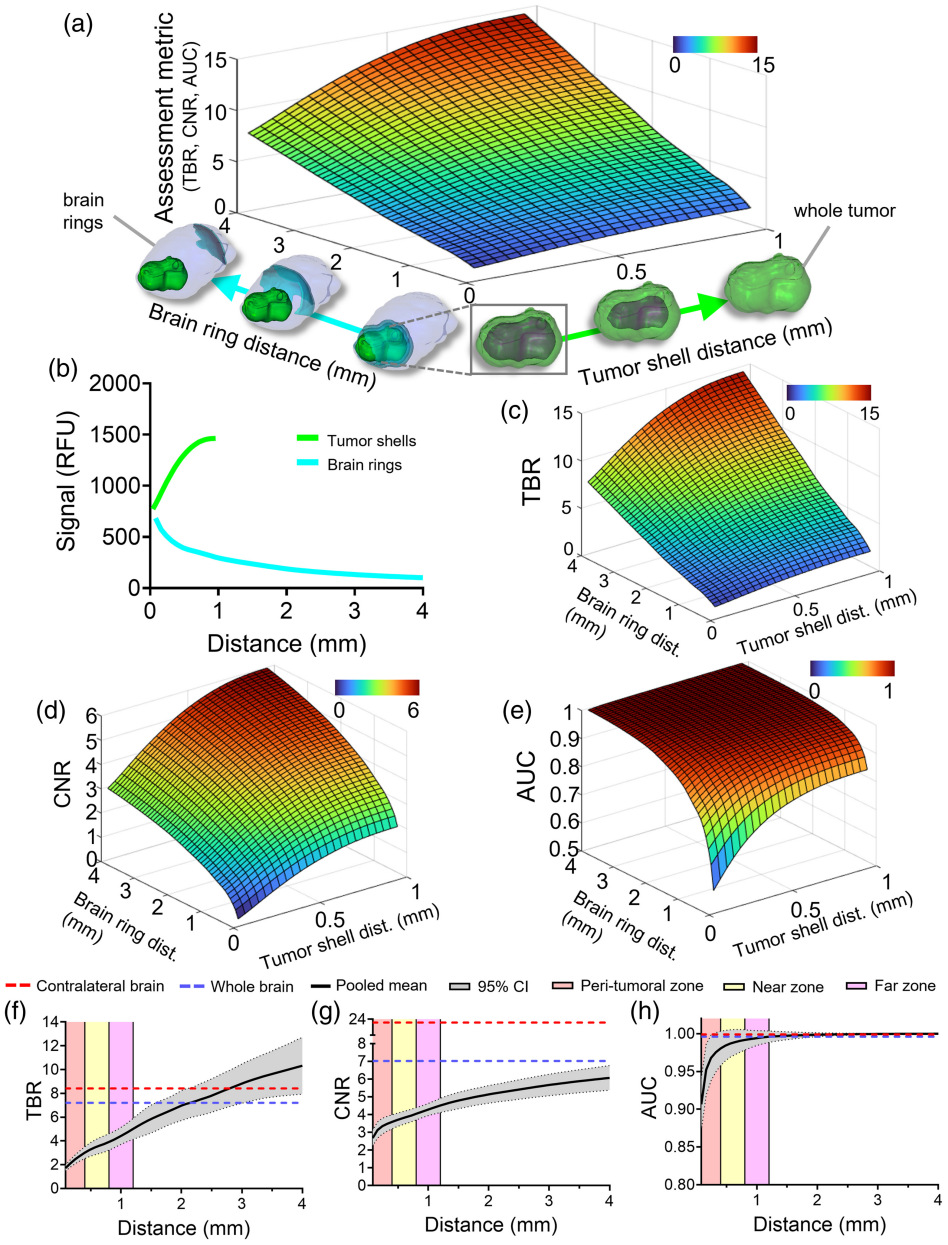
Results for the brain ring method. (a) Illustrative surface plot of a generic metric as the tumor and background regions are varied for a single animal. (b) Mean TMR-PEG1k signal intensity plotted as a function of distance for the tumor shells and brain rings. (c) Tumor-to-background (TBR) contrast ratio. (d) Contrast-to-noise (CNR) ratio. (e) Area under the receiver operating characteristic curve (AUC). (f)–(h) Contrast metric results for all animals using the whole tumor but increasing the brain ring distance: (f) TBR, (g) CNR, and (h) AUC. Data for panels (f)–(h) are presented similarly as described in Fig. 2.

Figure 3 has an identical structure to Fig. 2 but shows representative contrast metric data using the brain ring method for the same representative animal. This method is illustrated in the x- and y-axes of the 3D plot in Fig. 3(a). The tumor region changes identically to Fig. 3(a), but in the brain ring method, the defined background brain starts as an 80-μm-thick ring which then expands into the normal brain with each distance iteration. Figure 3(b) shows a plot of the mean TMR-PEG1k fluorescence intensity of the defined tumor and brain ring regions for each respective distance iteration for the same animal used in Figs. 2(a)–2(e). Inspection of Fig. 3(b) reveals that as the distance of the brain ring increases from the tumor border into the normal brain the brain ring mean intensity substantially decreases by ∼7× from the first to last ring.

Figure 3(c) shows the TBR plot and, similar to the brain dilation method, TBR values become elevated as the tumor shells include more of the tumor core irrespective of the brain ring that is being evaluated. In addition, as the distance of the defined brain ring region increases further from the tumor border the TBR values become elevated regardless of the tumor shell used. In general, using the brain ring method, the highest values are seen when the whole tumor and final brain ring iteration are used (TBR=14.4 for this animal). The CNR plot in Fig. 3(d) displays similar results to the TBR plot where CNR values increase when the tumor shell and brain ring distance from the tumor are increased. AUC values also increase rapidly to values near 1 as the distance increases [Fig. 3(e)].

The pooled assessments for all animals using the brain ring method are provided in Figs. 3(f)–3(h). The contralateral and whole brain-defined metrics, represented as the red and blue horizontal dotted lines, are also included. On each graph, the pooled mean is again displayed up to a distance of 4 mm (individual plots are provided in Fig. S3 in the Supplementary Material). As the brain ring expands, the TBR surpasses both the whole and contralateral brain methods reaching a peak mean of 10.3 at the final brain ring. The CNR data, provided in Fig. 3(g), show a minimum mean value of 2.7 with the peak mean value observed at the final iteration (CNR=6.1). All mean CNR values across this range were lower when compared with both the contralateral and whole brain-defined methods (20.0 and 7.0, respectively). Figure 3h shows the combined AUC results for the brain ring method. The brain ring method started to provide higher means than the whole brain (AUC=0.996) and contralateral brain methods just beyond the far zone of the background brain which persisted further into the normal brain. The lowest value was also seen here using the first 80  μm brain ring (AUC=0.907), and as the distance of the ring increased further into the normal brain, the AUC increased to 0.999 at 4 mm.

**Fig. 4:**
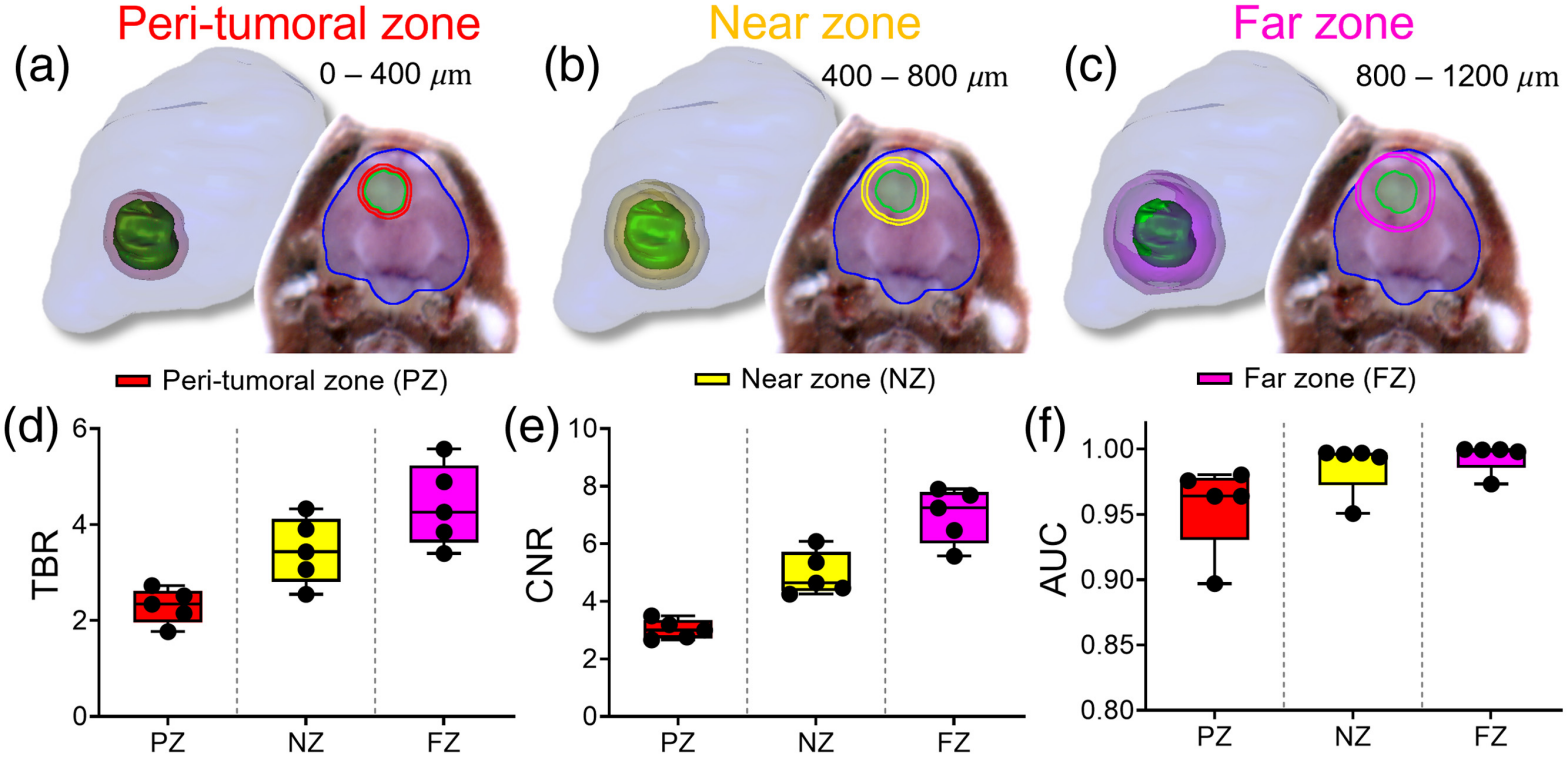
Contrast metric assessment using the peri-tumoral, near, and far zones. (a)–(c) Example showing the peri-tumoral (PZ), near (NZ), and far zones (FZ), respectively. (d)–(f) TBR, CNR, and AUC brain zone analysis results, respectively.

Figure 4 displays the methodology and results for the brain zone method. The three zones are illustrated in Figs. 4(a)–4(c) as rendered 3D surfaces and as a 2D cross-section. Generally, as the defined background zones move further from the tumor boundary and into the normal brain the assessed metrics improve. TBR results are provided in Fig. 4(d). All zones provide a mean TBR>2, but the far zone provided the highest TBR value (TBR = 4.4) followed by the near and peri-tumoral zones (TBR=3.5 and 2.3, respectively). CNR results are shown in Fig. 4(e) and display similar trends to the TBR plot where the highest CNR was calculated using the far zone and the near and peri-tumoral zones followed with all mean values >3 (CNR=7.0, 5.0, and 3.0, respectively). Figure 4(f) depicts the AUC results within these three cumulative zones. All zones provide an AUC>0.95, but the highest AUC is seen using the far zone as the background brain region (AUC=0.994).

## 4 Discussion and Conclusion

This study aimed to explore the effect that using different ROIs has on the performance metrics used to assess fluorescence imaging *in vivo*, with a particular focus on tumor imaging. Specifically, we used high-resolution 3D imaging of whole mouse heads bearing brain tumors to examine the TBR, CNR, and AUC of a fluorescent agent while methodically varying background and tumor ROIs over a wide range. Although others have reported performance metrics for different tumor and background regions which range from contralateral to near tumor margin ROI methods,^7^^,^^9^^,^^11^^,^^34^^,^^48^^–^^52^ to our knowledge, this is the most comprehensive analysis to date.

Our findings show that ROI selection has a major impact on the reported values of metrics used to evaluate fluorescence imaging strategies. The use of contralateral brain ROIs, which are representative of the ROIs most frequently used in the field, consistently produced the highest performance metrics. These metrics decreased dramatically as background ROIs approached or included regions adjacent to the tumor boundary, a consequence of fluorescence observed in the background brain in tumor-adjacent tissues. TBR, the most commonly used metric, changed by a factor of 5, CNR by a factor of 7, and AUC by over 10%, largely depending on the proximity of the background region to the tumor. Although these results may be self-evident, the magnitude of this effect has not been fully characterized before.

These results demonstrate the importance of careful ROI selection and of clearly reporting the regions used in publications. Although common, the use of contralateral background ROIs may dramatically overestimate the performance of the agent for a given application. The ideal ROI selection will likely vary across applications. For example, surgical guidance applications that involve tumor removal will likely require exquisite diagnostic performance in the peritumoral regions, whereas other applications, such as imaging nerve-specific or vascular agents, may not require such strict assessment of adjacent tissues. Understanding the desired clinical/study outcomes for the application, combined with studies similar to this one, can help guide ROI selection for a given application.

This study considered an orthotopic brain tumor model to examine the ROI selection effect, and thus, it is not a comprehensive assessment of the range of applications for which fluorescence guidance may be used. Although we do not anticipate the absolute values to be directly applicable to other tumors/agents/organs/applications, we believe the general trends are informative and should focus attention on the need for clarity around ROI selection. A potential limitation involves the use of GFP images for establishing ground truth. Although there can be some uncertainty in the precise tumor boundary, it is a laboratory standard for tumor tracking, and our use of the co-registered H&E slides for each animal further refined the tumor boundary selection.

The fluorescence cryotomography strategy used here isolates the fluorescent agent distribution and thus largely eliminates many of the challenging factors encountered during surgery, such as sub-surface fluorescence, nonuniform illumination, whole blood, and other confounders. Thus, the analyses herein focus primarily on the fluorescence distribution itself and can be thought of as bridging the gap between phantom studies and clinical studies. Examining this effect under these controlled conditions can be an important component in understanding contrast agent performance, and the fact that the metrics were impacted to such a large extent even under these conditions is notable.

The results herein show that ROI selection has a significant effect on evaluation metrics commonly used for fluorescence imaging strategies. As a practical guideline, future preclinical/clinical studies that aim to assess new agents and/or imaging strategies should carefully consider various ROI selections and at a minimum clearly report the ROIs used.

## Supplementary Material

10.1117/1.JBO.30.4.046004.s01

## Data Availability

All research data are available at (https://doi.org/10.6084/m9.figshare.c.7731755.v1).
