# Elucidating the effect of tumor and background region-of-interest selection on the performance metrics used to assess fluorescence imaging (Erratum)

**DOI:** 10.1117/1.JBO.30.5.059801

**Published:** 2025-05-23

**Authors:** Augustino V. Scorzo, Caleb Y. Kwon, Rendall R. Strawbridge, P. Jack Hoopes, David W. Roberts, Scott C. Davis

**Affiliations:** a Dartmouth College, Thayer School of Engineering, Hanover, New Hampshire, United States; b Dartmouth College, Geisel School of Medicine, Hanover, New Hampshire, United States; c Norris Cotton Cancer Center, Dartmouth-Hitchcock Medical Center, Lebanon, New Hampshire, United States

## Abstract

The erratum corrects an error in the originally published article, adding a citation.

This article [*J. Biomed. Opt.* **30**(4), 046004 (2025) doi: 10.1117/1.JBO.30.4.046004] was originally published on 2 April 2025 with a missing citation and a missing reference.

The article was corrected and republished 20 May 2025.

The original version did not include a citation for 3DSlicer when describing a rigid registration between the cryotomography and pathology images in Section 2.4 (though 3DSlicer was cited elsewhere in the paper).

The corrected version includes a citation to 3DSlicer when introducing the registration process in Section 2.4, as well as a citation to the following PhD thesis that used rigid registration to register similar types of images:

B. K. Byrd, “Advancing fluorescent contrast agent recovery methods for surgical guidance applications,” PhD Dissertation, Dartmouth College, p. 151 (2022).

