# Supplementary material for: Elucidating the effect of tumor and background region-of-interest selection on the performance metrics used to assess fluorescence imaging
Source: J Biomed Opt. 2025 Apr 2;30(4):046004. doi: 10.1117/1.JBO.30.4.046004 (PMC11963147; doi:10.1117/1.JBO.30.4.046004)
Supplement: Supplementary file 1 [file JBO_030_046004_SD001.docx]

# Supplemental Material


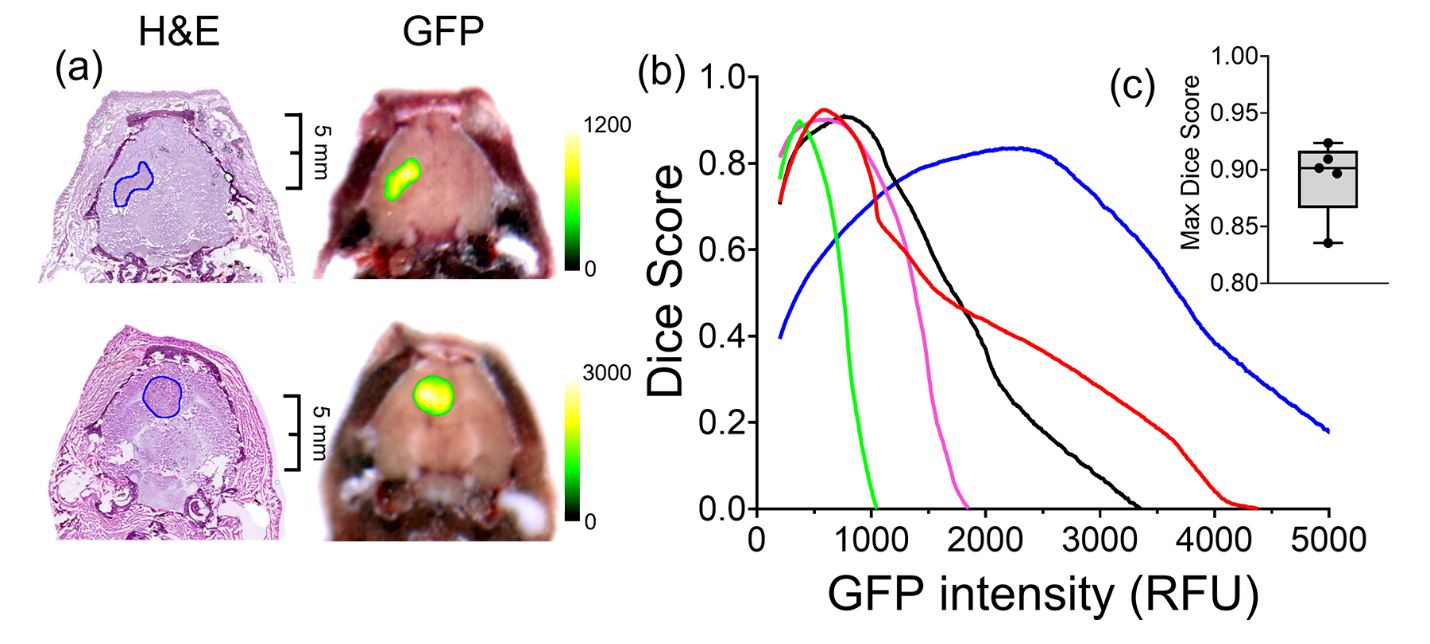


Fig. S1 Methodology for tumor ROI determination based on histopathology. **(a)** Co-registered H&E (with the tumor outlined in blue) and GFP fluorescence measured in relative fluorescence unites (RFU). **(b)** Dice similarity scores between co-registered H&E and GFP fluorescence as a function of discriminating GFP intensity (RFU) for 5 mice (one animal for each color). **(c)** The maximum Dice score determined for each animal represented as a boxplot with median and interquartile range.


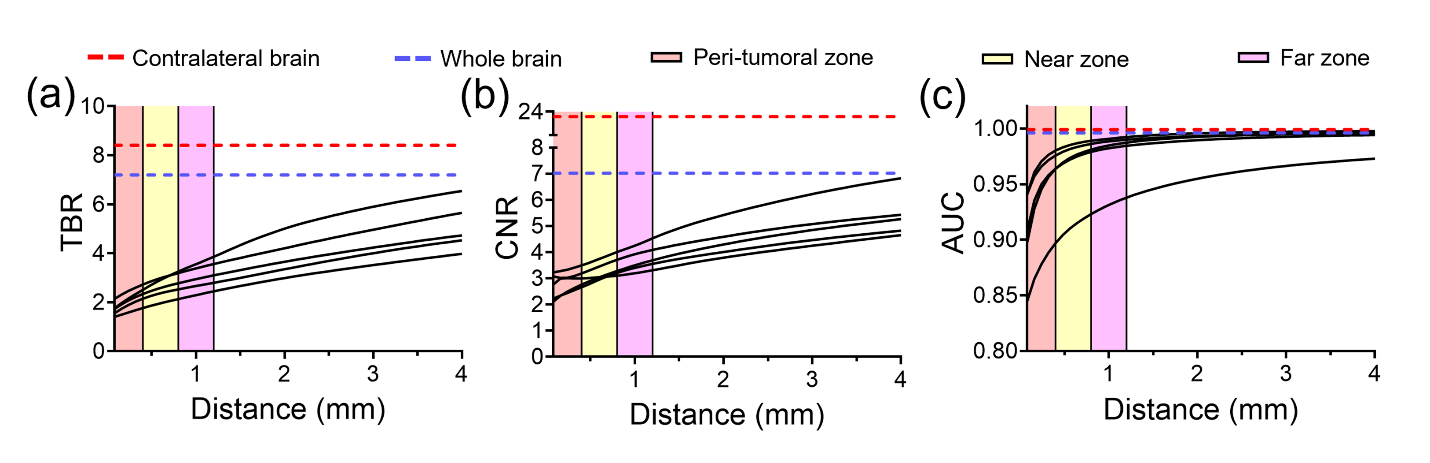


Fig. S2 Results for the brain dilation method for each individual animal. Each animal is represented by a solid black line. (a-c) Contrast metric results using the whole tumor but increasing the brain dilation distance: (a) TBR (b) CNR and (c) AUC. The pooled mean for each contrast metric using the whole brain and contralateral brain as the define background are represented as dotted lines on the plot and the peri-tumoral, near and far zone are represented as colored zones.


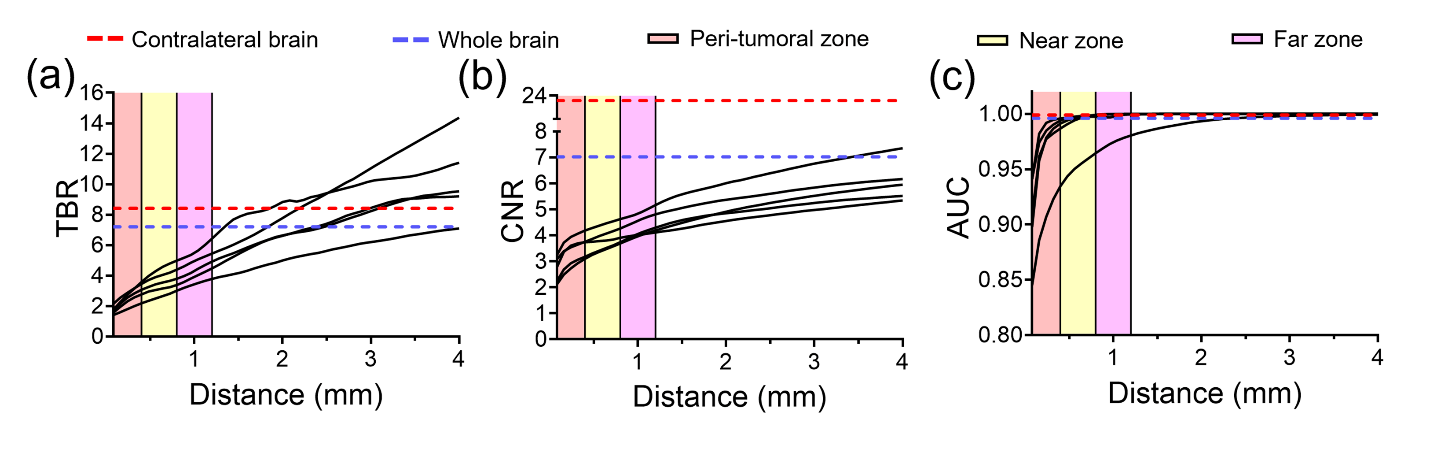


Fig. S3 Results for the brain ring method for each individual animal. Each animal is represented by a solid black line. (a-c) Contrast metric results for each individual animal using the whole tumor but increasing the brain ring distance: (a) TBR (b) CNR and (c) AUC. Contralateral brain, whole brain and brain zones are depicted identically as seen in Fig. S1.
